# Do Different High‐Intensity‐Focused Ultrasound Frequencies Have Different Effects? A Histological Analysis Correlated With Patients' Subjective Assessments

**DOI:** 10.1111/jocd.70069

**Published:** 2025-02-19

**Authors:** Yousun Hwang, Jovian Wan, Kyu‐Ho Yi

**Affiliations:** ^1^ Haedrin Clinic Center for Aesthetic Medicine Seoul Korea; ^2^ Medical Research Inc. Wonju Korea; ^3^ Division in Anatomy and Developmental Biology, Department of Oral Biology, Human Identification Research Institute, BK21 FOUR Project Yonsei University College of Dentistry Seoul Korea; ^4^ You & I Clinic (Mokdong) Seoul Korea

**Keywords:** aesthetic dermatology, collagen synthesis, facial contouring, high‐intensity‐focused ultrasound, skin rejuvenation

## Abstract

**Background:**

High‐intensity‐focused ultrasound (HIFU) has emerged as a noninvasive approach for facial rejuvenation, offering benefits such as collagen synthesis and subcutaneous fat reduction. However, the differential effects of varying HIFU frequencies on specific skin layers remain underexplored.

**Aims:**

To evaluate the histological effects of different HIFU frequencies and correlate these findings with patient‐reported outcomes, thereby optimizing clinical applications.

**Patients/Methods:**

Histological analysis was performed on pig skin treated with HIFU at specific depths (2.0, 3.0, 4.5, and 6.0 mm) using the Ultraformer MPT device. Collagen types I and III, elastin fibers, and fat reduction were assessed using immunohistochemistry, Victoria blue staining, and Oil Red O staining. Additionally, 50 Asian female participants aged 30–60 years with skin laxity underwent HIFU treatment. Clinical outcomes were evaluated using standardized photographs, the Global Aesthetic Improvement Scale (GAIS), and a modified Rosenberg Self‐Esteem Scale.

**Results:**

Histological analysis revealed increased collagen and elastin fiber synthesis and significant fat reduction post‐HIFU. Clinical outcomes corroborated these findings, with 85.3% of participants reporting overall satisfaction and 70.6% noting improvements in facial contouring. Self‐esteem assessments indicated a positive psychological impact, with 64.7% of participants expressing enhanced confidence.

**Conclusions:**

HIFU treatment at varying frequencies induces significant histological and clinical improvements, demonstrating its efficacy for skin rejuvenation. This study underscores HIFU's dual role in enhancing physical appearance and psychological well‐being, supporting its integration into patient‐centric aesthetic care.

## 1 Introduction

The desire for beauty and youthfulness has been a defining pursuit throughout human history, with each era developing strategies to counter the visible signs of aging. In modern times, advances in medical science have led to remarkable improvements in life expectancy, which now averages over 79 years in Organization for Economic Co‐operation and Development countries. As individuals enjoy longer, healthier lives, the demand for maintaining a youthful appearance has intensified, resulting in a rise in both surgical and nonsurgical cosmetic procedures [1, 2].

Among the many noninvasive aesthetic treatments, high‐intensity‐focused ultrasound (HIFU) has gained prominence as an effective modality for facial rejuvenation. HIFU's ability to stimulate collagen synthesis and reduce subcutaneous fat has been well‐documented, with studies demonstrating improvements in skin laxity and facial contour [3, 4, 5, 6, 7]. Histological evidence also supports HIFU's capacity to increase collagen fiber production, contributing to its efficacy in skin tightening [8, 9]. However, the specific effects of varying HIFU frequencies on different skin layers remain under explored.

This study aims to address this gap by investigating the histological changes induced by different HIFU frequencies. Additionally, we seek to correlate these findings with subjective assessments from participants, aiming to optimize HIFU protocols for personalized patient care. We also explore the potential impact of HIFU on participants' confidence levels, recognizing the broader psychological effects of cosmetic treatments.

Changes in facial appearance, whether due to aging or trauma, are known to significantly impact self‐esteem and body image. In a society increasingly focused on aesthetic ideals, these concerns can lead to psychological distress [10, 11]. As cosmetic procedures become more normalized, healthcare providers must remain mindful of the psychological implications for patients whose appearances deviate from perceived norms.

In this context, visible differences in appearance can create psychosocial challenges, often linked to skin conditions that exacerbate psychological difficulties [12]. This study also examines the relationship between perceived confidence and the choice of cosmetic interventions, with a particular focus on the psychological outcomes of HIFU treatment.

Given the growing importance of physical appearance in contemporary society, cosmetic treatments are often seen as pathways to enhanced self‐esteem. However, they can also foster a reliance on aesthetic interventions. This investigation seeks to contribute to a more nuanced understanding of the role of noninvasive aesthetic treatments, offering insights into the psychological and social dimensions of cosmetic procedures.

## 2 Method

In this study, we utilized the skin of a crossbred female pig harvested from a deceased animal following ethical guidelines for tissue donation to examine the production of elastin fibers, collagen types I and III, and fat cell reduction following treatment with a HIFU device (Ultraformer MPT, Classys Inc.). The tissue was collected immediately after the animal's death and processed within an hour, ensuring ethical transparency and reproducibility. The Ultraformer MPT is the latest HIFU device from Classys, incorporating a micro‐pulsed mode. This mode features microspacing between Thermal Coagulation Point (TCP) sites, enabling more efficient energy delivery in a single shot. Each cartridge is designed to target specific tissue depths (2.0, 3.0, 4.5, and 6 mm). After administering one shot (0.1 s) to the pig skin placed on a heated plate to simulate the temperature of live human skin, we performed immunohistochemical staining on biopsied skin samples to evaluate collagen types I and III production. Elastin fiber synthesis was assessed using Victoria blue staining, and fat reduction was determined through Oil Red O staining.

Post‐HIFU tissue samples were stored in DMEM (Dulbecco's Modified Eagle Medium) at 37°C with 5% CO2 and 95% humidity for 48 h to allow for collagen synthesis, elastin fiber regeneration, and fat reduction. This environment was chosen to replicate in vivo conditions as closely as possible, ensuring that the tissue would remain viable for histological analysis. Immediate fixation was applied using 10% formalin following this period to preserve tissue integrity and support the study's goals.

To correlate the histological findings with clinical outcomes in human subjects, we conducted a study involving 50 Asian females, aged 30–60 years (Fitzpatrick skin types III and IV), all presenting with mild to severe skin laxity. Exclusion criteria included active infections or skin diseases, pregnancy, a history of keloidal scarring, and any anti‐aging procedures within the previous 12 months. Informed consent was obtained from all participants at the time of enrollment. Following facial cleansing, a topical anesthetic cream (5% lidocaine‐prilocaine) was applied to the entire face for 40 min.

HIFU treatment was administered using 300 shots per participant (150 shots at a depth of 3.0 mm and 150 shots at 4.5 mm). The 4.5 mm cartridge was used exclusively on the lower face, while the 3.0 mm cartridge targeted the midface and periocular regions. The energy settings were 1.0 J for the 4.5 mm cartridge and 0.3 J for the 3.0 mm cartridge. Standardized digital photographs were captured before and 3 months after treatment under identical lighting conditions. Images were taken from the front and at a 45° angle on both sides.

The pre‐ and posttreatment photographs were presented to a blinded clinician, who was asked to identify the posttreatment image and assess the overall improvement using the Global Aesthetic Improvement Scale (GAIS). The GAIS ratings ranged from (1) very much improved to (5) worsened. Participants were also asked to rate their overall improvement and specifically evaluate skin tightening and facial contouring using the same scale.

To further assess psychological outcomes, we explored whether the participants' self‐esteem had improved following treatment. Using a modified version of the Rosenberg Self‐Esteem Scale, we asked participants to respond to two key statements: (1) whether they were more satisfied with themselves and (2) whether they adopted a more positive attitude toward themselves.

## 3 Results

**FIGURE 1:**
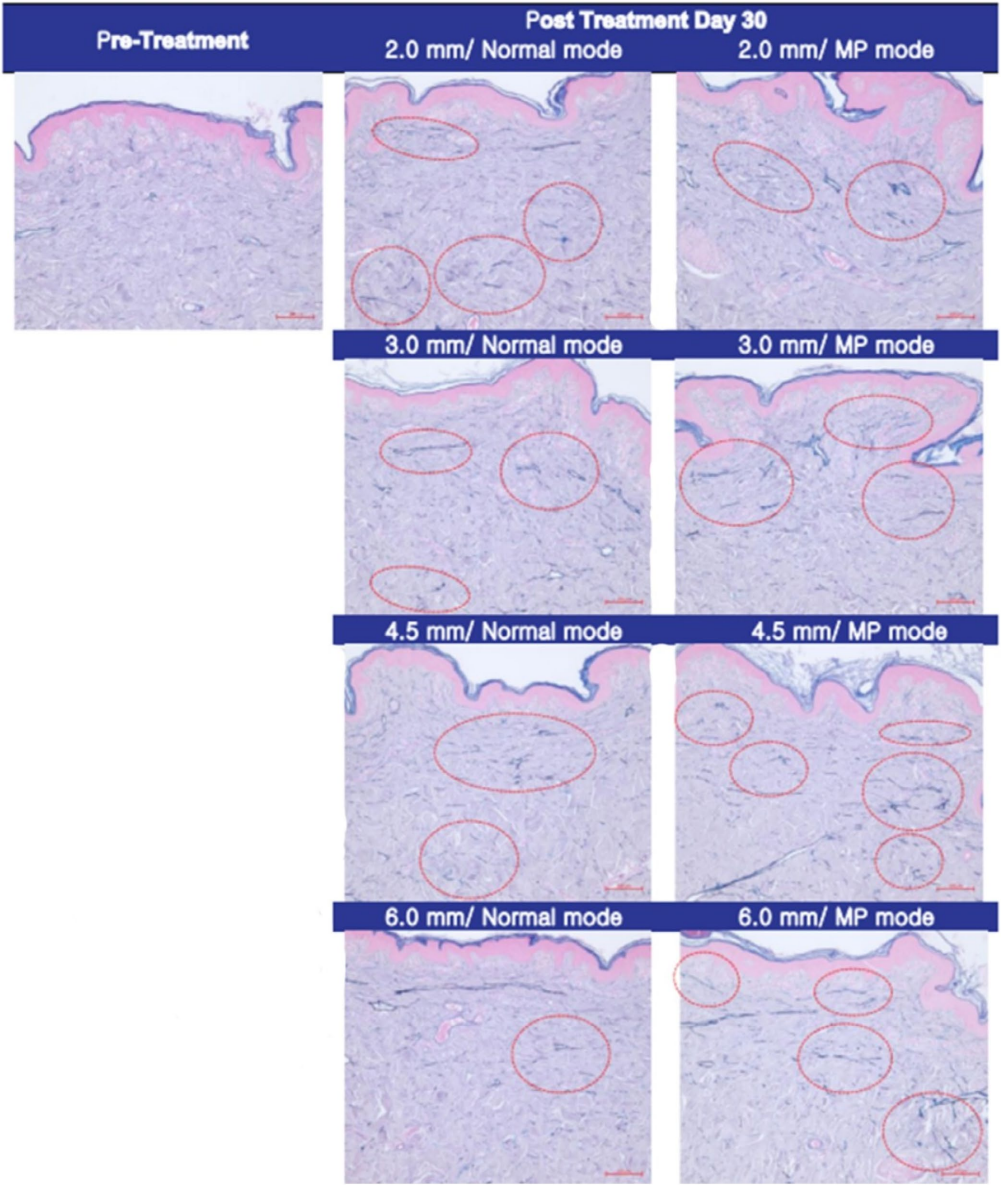
Victoria blue staining 30 days post‐HIFU treatment. The regeneration of elastin fibers is clearly visible as red circles within the deep dermis, indicating a response to the thermal injury caused by HIFU treatment. These observations suggest tissue remodeling and skin tightening effects over the course of the healing process (magnification ×100, scale bar = 100 μm).

**FIGURE 2:**
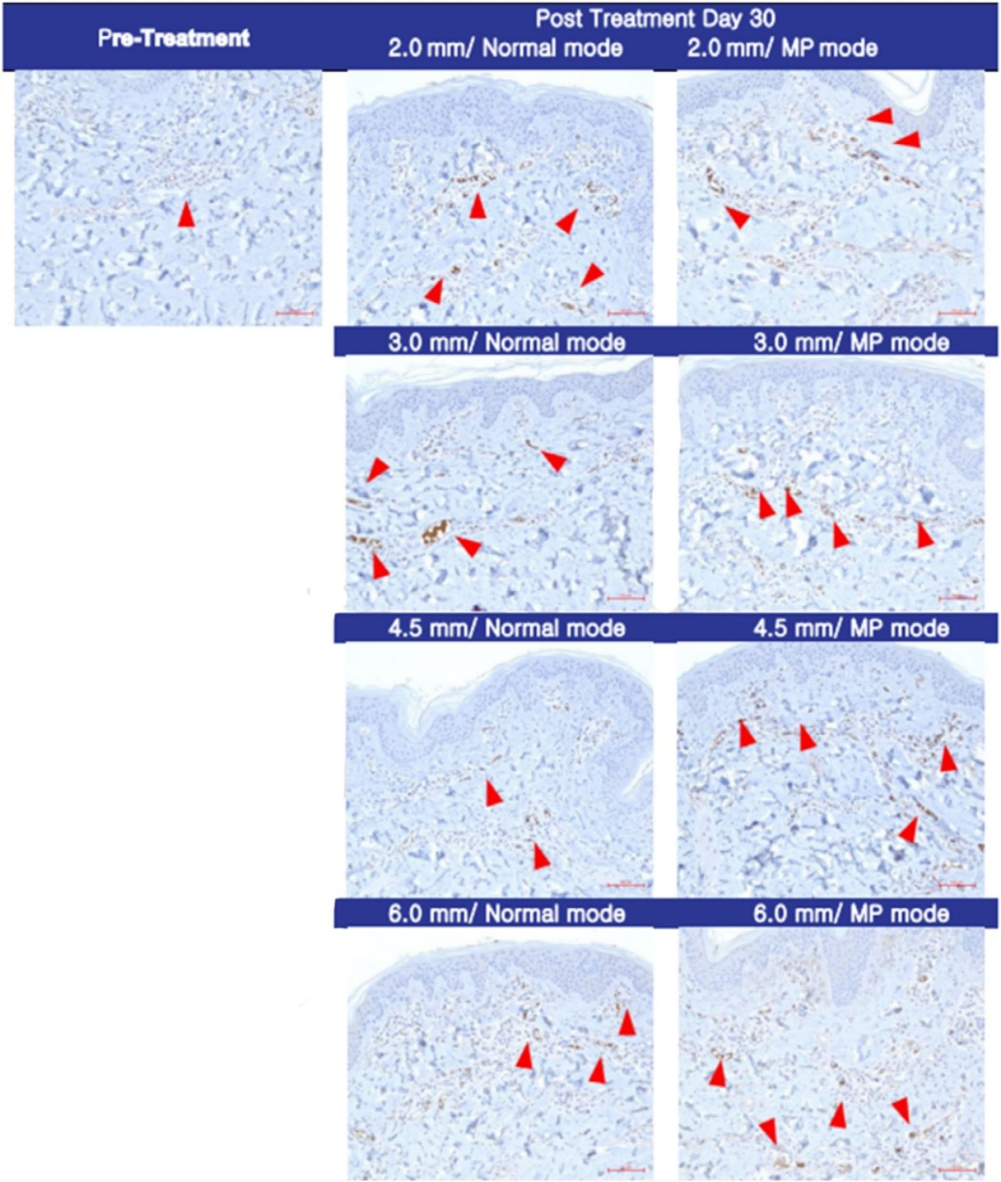
Immunohistochemical staining 30 days post‐HIFU treatment. The synthesis of new collagen, including types I and III, is indicated by red arrows. This highlights the collagen remodeling response triggered by HIFU treatment, which is crucial for improving skin elasticity and reducing signs of aging (magnification ×200, scale bar = 50 μm).

**FIGURE 3:**
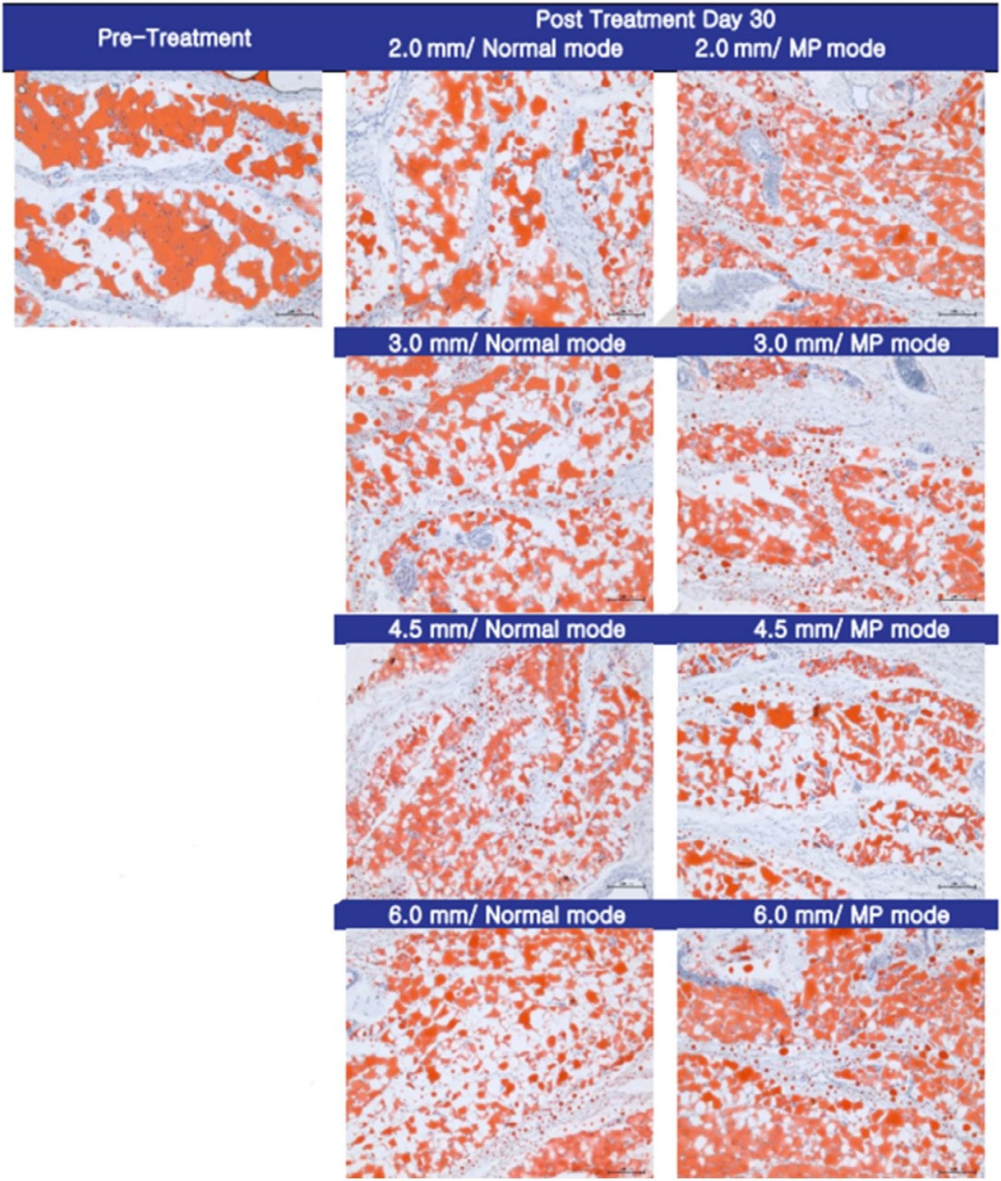
Oil Red O staining 30 days post‐HIFU treatment. A significant reduction in fat in the treated areas is observed, demonstrating the fat cell destruction induced by HIFU. This fat reduction contributes to the skin tightening and contouring effects associated with the procedure (magnification ×200, scale bar = 50 μm).

Histological analysis of pig skin samples 30 days post‐HIFU treatment revealed significant tissue regeneration. Victoria blue staining (Figure 1) demonstrated the regeneration of elastin fibers, visualized as red circles, while immunohistochemical staining (Figure 2) confirmed new collagen synthesis, indicated by red arrows. Oil Red O staining (Figure 3) further showed a marked reduction in subcutaneous fat in the treated areas.

These findings were consistent with the clinical outcomes observed in human participants. HIFU treatment induced a notable increase in neocollagenesis and neoelastogenesis within the deep dermis. Immunohistochemical staining and Victoria blue staining confirmed the synthesis of collagen types I and III, as well as elastin fibers. Additionally, Oil Red O staining revealed significant fat reduction, corroborating the histological evidence of fat cell destruction.

Out of the 50 participants initially enrolled in the study, 34 completed the full treatment and follow‐up. The blinded clinician was able to correctly identify posttreatment photographs in 73.5% of cases (25/34), further validating the visible changes induced by HIFU. In terms of participant satisfaction, 85.3% (29/34) of subjects reported being pleased with the overall results of their HIFU treatment. When specifically asked about facial slimming and skin tightening, 70.6% (24/34) and 64.7% (22/34) of participants, respectively, expressed satisfaction with the outcomes. Furthermore, 64.7% (22/34) of participants reported a more positive attitude toward themselves following the treatment, with 61.8% (21/34) stating they were generally more satisfied with their appearance.

## 4 Discussion

Over the past several decades, significant advancements have been made in addressing facial aging through medical interventions. Among the various emerging treatments, HIFU has gained recognition as one of the most effective methods for improving mild to moderate skin laxity. Its effects on facial contouring and skin tightening have been well‐documented in the literature [13].

HIFU operates by delivering focused, high‐intensity ultrasonic waves to targeted depths within the skin. The thermal energy and mechanical pressure generated by these waves create thermal injury points, which induce fat cell destruction, collagen denaturation, and subsequent neocollagenesis [14]. There have been several attempts to demonstrate histological changes following HIFU treatment. For instance, Suh et al. [15] observed significant collagen synthesis in the reticular dermis after HIFU application. This study builds on Suh's findings by also verifying fat cell destruction in pig skin samples. While pig skin shares physiological and histological similarities with human skin, it should be noted that there are inherent species differences that limit the direct extrapolation of these findings to humans. However, several key histological and physiological similarities make the pig model particularly relevant for this type of research [16]. Pig skin shares several structural and functional characteristics with human skin, including similar epidermal thickness, collagen structure, and fat layer composition, which are critical in evaluating the effects of HIFU treatment [17]. Specifically, pig skin exhibits similar dermal collagen organization and responds to thermal injury with comparable neocollagenesis and fat cell reduction, making it a reliable surrogate for human skin in this study [16, 18].

Additionally, the fat distribution in pigs is similar to that of humans, with subcutaneous fat located in comparable anatomical regions, which allowed for an accurate assessment of the fat reduction effects of HIFU. This physiological similarity enhances the relevance of our histological findings in the pig model, particularly when evaluating the thermal injury points and their effects on collagen and fat [19, 20].

Furthermore, previous studies have demonstrated the successful extrapolation of pig model results to human clinical outcomes [21, 22]. For example, research in aesthetic treatments, such as laser and HIFU therapies, often utilizes pig models due to their high degree of physiological resemblance to humans, particularly in the response of skin to thermal and mechanical stimulation. These studies have consistently shown that histological findings in pig skin, such as collagen synthesis and elastin regeneration, are reflective of what occurs in human skin following similar treatments [23, 24].

In our study, the histological analysis of pig skin samples showed significant collagen production, elastin fiber regeneration, and fat reduction, which were consistent with the clinical outcomes observed in human participants. The presence of neocollagenesis and elastogenesis in both the pig model and human participants supports the validity of the findings and enhances the relevance of our pig model results to human applications. Additionally, the improvements in skin tightening and facial contouring observed in human participants align with the histological changes seen in the pig skin samples, further reinforcing the translational potential of our results.

In the majority of cases, both the blinded physician and the participants reported noticeable improvements following HIFU treatment. These positive changes, such as visible skin tightening and slimming effects, align with the histological changes observed, further supporting the efficacy of HIFU. Additionally, self‐esteem assessments revealed that most subjects reported feeling better about themselves after treatment. Body image and self‐esteem are closely linked to subjective well‐being. Studies consistently show that negative perceptions of appearance are associated with low self‐esteem and various psychological disorders [25, 26, 27]. Conversely, a positive body image has been shown to enhance psychological well‐being [28]. In a society that places significant emphasis on physical appearance, this correlation between self‐esteem and appearance can pose challenges to mental health, particularly for women. Numerous young women are vulnerable to conditions such as eating disorders and body dysmorphic disorder, often exacerbated by social media exposure to idealized images of celebrities and influencers. On the other hand, middle‐aged women tend to respond differently to societal pressures; middle‐aged women often experience feelings of loss related to aging, compounded by hormonal changes [29]. However, they tend to have a more realistic understanding of the limitations of cosmetic interventions, often opting for less aggressive treatments to enhance their appearance [30].

The improvement in confidence observed among participants following Ultraformer III HIFU treatment underscores the dual benefits of this procedure: not only enhancing physical appearance but also alleviating the psychological distress associated with aging. Confidence levels were assessed alongside satisfaction rates, with a significant increase in self‐esteem noted after treatment. This finding highlights the potential of noninvasive cosmetic procedures like HIFU to effectively address the self‐esteem concerns of middle‐aged women, demonstrating the broader emotional benefits of such treatments.

Folliscope analysis further validated the precision of the HIFU treatment. For instance, thermal injury points created by the 1.5 mm cartridge were accurately positioned at the targeted depth of 1.5 mm from the skin surface. Additionally, the area of TCPs generated by the micro‐pulsed mode was significantly larger than that produced by the normal mode. These findings confirm that the thermal energy delivered by HIFU is effectively targeting the intended depths, thereby optimizing treatment outcomes and enhancing safety.

In conclusion, the results of this study emphasize the comprehensive benefits of Ultraformer III HIFU treatment in addressing both the physical and psychological aspects of aging in middle‐aged women. The observed histological changes, such as collagen synthesis and fat cell destruction, correlate with the subjective improvements in skin tightening and facial contouring reported by participants. Moreover, the notable boost in self‐esteem and confidence underscores HIFU's potential as a valuable tool for enhancing overall well‐being.

By better understanding the expected outcomes of HIFU, both physicians and patients can make more informed decisions, set realistic expectations, and maximize the positive effects of the procedure. This study contributes to the growing body of evidence supporting noninvasive cosmetic treatments, not only for physical enhancement but also for addressing the emotional and psychological challenges of aging. As the field progresses, it is vital to adopt a holistic approach to beauty and well‐being, recognizing that true confidence arises from more than just physical appearance.

## Author Contributions

All authors have reviewed and approved the article for submission. Conceptualization: Yousun Hwang. Study design: Yousun Hwang, Jovian Wan, and Kyu‐Ho Yi. Acquisition of data, Yousun Hwang. Writing – original draft preparation: Yousun Hwang and Jovian Wan. Writing – review and editing: Jovian Wan and Kyu‐Ho Yi. Visualization: Yousun Hwang. Supervision: Kyu‐Ho Yi.

## Ethics Statement

The study adhered to the principles of the Declaration of Helsinki, and Institutional Review Board review was deemed unnecessary and waived for this study due to the nature of the investigation.

## Consent

Informed consent was obtained from all subjects involved in the study. Written informed consent was obtained from the patients to publish this paper.

## Conflicts of Interest

The authors declare no conflicts of interest.

## Data Availability

The data that support the findings of this study are available from the corresponding author upon reasonable request.
